# Influence of orbital morphology on proptosis reduction and ocular motility after decompression surgery in patients with Graves’ orbitopathy

**DOI:** 10.1371/journal.pone.0218701

**Published:** 2019-06-24

**Authors:** Michael Oeverhaus, Anna Copei, Stefan Mattheis, Adrian Ringelstein, Madeleine Tiemessen, Joachim Esser, Anja Eckstein, Kerstin Stähr

**Affiliations:** 1 Department of Ophthalmology, University Hospital Essen, Essen, Germany; 2 Department of Gynecology, University Hospital Oldenburg, Oldenburg, Germany; 3 Department of Oto-Rhino-Laryngology, Head and Neck Surgery, University Hospital Essen, Essen, Germany; 4 Department of Radiology, Maria Hilf Hospital, Mönchengladbach, Germany; 5 Department of Radiology and Neuroradiology, Alfried Krupp Hospital Essen, Essen, Germany; Faculty of Medicine, Cairo University, EGYPT

## Abstract

**Purpose:**

Orbital decompression surgery is performed in patients with Graves’ orbitopathy to treat dysthyroid optical neuropathy (DON) and reduce disfiguring proptosis. The intended proptosis reduction can deviate from the postoperative result and changes of motility with consecutive diplopia can occur. We performed a retrospective study to identify anatomical factors in computed tomography (CT), which influence the surgical effect and postoperative ocular motility and diplopia.

**Methods:**

Pre- and postoperative CT-scans of 125 eyes of 68 patients, who mainly underwent a balanced orbital decompression for disfiguring proptosis (≥18mm Hertel Index), have been analyzed. Proptosis, ductions, misalignment and diplopia were assessed before and after surgery. Medial and lateral orbital wall length, conus angle, depth of ethmoidal sinus, orbital surface, length of medial and orbital defect, depth of tissue prolapse and horizontal muscle diameters were analyzed in CT scans before and after surgery. With linear regression and multivariate analyses these parameters have been correlated with postoperative proptosis, abduction deficit, deviation and binocular single vision (BSV).

**Results:**

Proptosis could be reduced by 5.3±2mm. Patients with <5mm proptosis reduction had significantly less often new onset of diplopia compared to patients with >5mm reduction (13% vs. 56%, p = 0.02). Multiple linear regression showed a significant correlation between tissue prolapse and depth of the ethmoidal sinus as well as age (p<0.001, r = 0.71). Proptosis reduction could not be predicted by tissue prolapse, defect length or depth of ethmoidal sinus. The abduction deficit correlated significantly with tissue prolapse and orbital surface area (p<0.001, r = 0.37) but not with the horizontal muscle diameter.

**Conclusion:**

We were able to show that orbital morphology influences the outcome of balanced orbital decompression surgery in terms of proptosis reduction and motility. However, the rather low coefficients of correlation show that the surgical outcome cannot be predicted with simple CT measurements, although risk factors for postoperative abduction deficit could be found. Therefore, preoperative planning should consider especially the orbital surface area and depth of ethmoidal sinus. Patients should be informed about the higher risk of diplopia with higher proptosis reduction.

## Introduction

Graves’ orbitopathy (GO) is the most common extrathyroidal manifestation of Graves’ disease. Mild stages present with subtle clinical features which are highly variable and often lead to misdiagnosis [1]. Autoimmune triggered inflammation leads typically to pain, swelling of soft orbital tissues, diplopia and eyelid retraction (due to enlargement and fibrosis of ocular muscles) and proptosis (due to adipogenesis) [2, 3]. Especially, proptosis and diplopia deteriorate the quality of life of affected patients [4–7]. During the last several decades, the underlying pathophysiology has been elucidated [3, 8–10]. However, targeted therapies (like Teprotumumab) are still undergoing clinical trials, which is why intravenous glucocorticoids (GCs) and orbital irradiation (ORT) comprise first-line treatment for moderate-to-severe disease stages, until newly developed specific therapies are available [11–16].

Due to the enlargement of extraocular muscles and adipogenesis in the fixed volume of the bony orbit, compression of the optic nerve can occur. This dysthyroid optic neuropathy (DON) leads to visual impairment, color vision loss and ultimately, if untreated, to blindness [1, 17]. Patients with such a sight-threatening GO require high-doses of intravenous GCs and surgical intervention [18, 19]: Bony decompression surgery can enlarge the orbital volume, lower the intraorbital pressure and thereby prevent functional impairment [18, 20, 21]. Orbital decompression can also be performed as rehabilitative surgery to reduce disfiguring proptosis, once a stable inactive GO and euthyroidism are achieved [22]. Further rehabilitative surgical interventions (e.g. squint and eyelid surgery) should follow after decompression surgery, since globe position, rotation, alignment and relative relationship to periocular soft tissue structures might be altered [18, 23, 24]. Depending on the desired effect, the lateral, medial and inferior wall can be resected, which enables expansion of the soft tissue and thereby reduction of orbital compression [18]. In addition, orbital fat can be removed to increase this effect [25]. Since Dollinger (1911) first described the orbital decompression, numerous different techniques and approaches have been developed with variable amounts of proptosis reduction and complication rates [18, 20, 25–32]. Recent data shows that minimal invasive and endoscopic approaches are associated with lower complication rates and greater efficacy [20, 25, 27, 33]. However, given the intricate osseous and soft tissue anatomy of the orbit, decompression surgery cannot be performed without risks. New onset or deterioration of strabismus can occur, especially after medial wall resection. This is due to the displacement of the medial rectus in the ethmoidal sinus, which also leads to an abduction deficit [34]. Patients might suffer consecutively from diplopia, which is severely decreasing quality of life [7].

Planning of orbital decompression involves mainly the desired proptosis reduction and the number of walls (one-, two-, three-wall surgery), but less the individual orbital morphology [27, 35]. The intended proptosis reduction can deviate from the postoperative result. Recently, single-center experiences with different three-dimensional planning approaches have been described. However, there has been no data on the efficacy of these techniques yet [36, 37].

We performed this retrospective analysis to evaluate the influence of individual anatomical features of the orbit on the outcome of decompression surgery in terms of proptosis reduction, eye motility and diplopia. Knowledge about influential factors might enable surgeons to individually tailor surgery. This could lead to less motility disorders and less variability in proptosis reduction. Therefore, we evaluated pre- and postoperative bony configuration of the orbit in computed tomography (CT) scans and correlated these data with ophthalmological examinations of patients who underwent bony decompression surgery in our tertiary referral center.

## Patients and methods

### Study population

We retrospectively analyzed patient records of the Essen EUGOGO (European Group On Graves’ Orbitopathy) and West German Orbital center for patients who underwent balanced decompression surgery. The study was performed under adherence of the ethical foundations of the Declaration of Helsinki and approved by the Ethics Commission of the University of Essen. Informed consent was not necessary because of the retrospective nature of this analysis. Only patients who met the following criteria were included: pre- and postoperative CT scan, complete ophthalmological examination pre- and postoperatively, proptosis ≥18mm, 18–80 years of age.

### Ophthalmological examination

Eye examinations were performed using a slightly modified EUGOGO case record form. First, all patients were evaluated by a highly trained orthoptist and afterwards by one of two specialized ophthalmologists (A.E., J.E.) before surgery. All patients underwent follow-up examinations 3 months after surgery by the same ophthalmologist, thereby securing homogeneity and reproducibility of the examination. GO was diagnosed in presence of typical clinical signs on ophthalmological examination, including slit-lamp biomicroscopy, applanation tonometry, funduscopy, Hertel and Naugel exophthalmometry, assessment of subjective diplopia and objective measurement of misalignment using the prism-cover-test, and measurement of monocular excursions and visual acuity. The field of binocular single vision was assessed using the Harms’ tangent scale at each visit. The assessed field was scored according to Sullivan et al. (1992) [38], who developed a scoring grid system. In this grid, the central areas of the field of binocular single vision and the down gaze are rated much higher since primary gaze and down gaze are most important for the quality of life. Patient experiencing no diplopia scored 100 points, double vision present in all gazes scored 0 points.

### Surgical procedure

**Fig 1:**
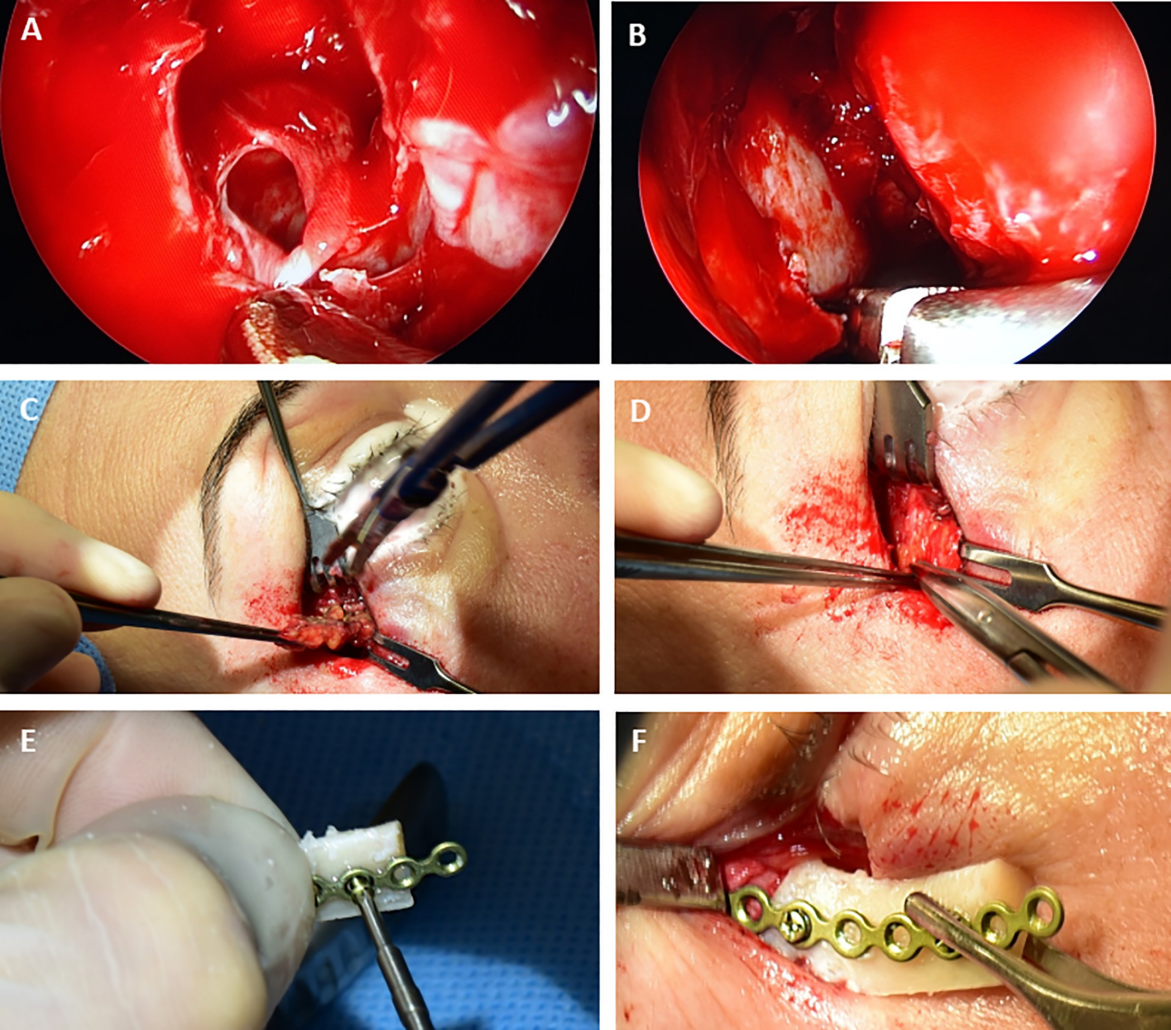
All patients underwent balanced orbital decompression. The extent of surgery was chosen according to the individual proptosis. **(A-B)** The medial wall was decompressed via endonasal endoscopy. The periorbit was incised allowing the orbital content to prolapse into the ethmoid cavity. **(C-D)** Resection of a bony triangle of the lateral wall was performed through a 10mm incision next to the lateral canthus. **(E-F)** To restore the lateral orbital rim, the anterior part of the resected bone is replanted employing a fixation with microplates.

All patients underwent surgery similar to a balanced orbital decompression as described before [39]. In brief, surgery was performed under general anesthesia. The extent of surgery was chosen according to the grade of proptosis [40]. The medial wall–and if necessary parts of the inferior wall–are decompressed via endonasal endoscopy (see Fig 1A and 1B). First, the uncinate process and the ethmoid are removed completely while preserving the medial turbinate. The skull base, the ostia of frontal and sphenoid sinus, lamina parpyracea and the inferior orbital wall are exposed. The lamina parpyracea is infractured and removed exposing the periorbita. In severe cases, the posterior medial part of the orbital floor is resected as well. The periorbit is incised from posterior to anterior while sparing the eye muscles and the optical nerve allowing the orbital content to prolapse into the ethmoid cavity. For resection of the lateral wall, a 10mm incision next to the lateral canthus is performed. The periosteum is dissected and a bony triangle of the lateral wall is removed (see Fig 1C and 1D). Remaining bone rims of the deep lateral wall are trimmed using a high-speed burr to create a smooth surface. After resecting the lateral periorbit 1.5–3 ml of orbital fat can be removed. To restore the lateral orbital rim, the anterior part of the resected bone is replanted employing a fixation with microplates (see Fig 1E and 1F). According to the concept of a graduated procedure, we resected the posterior part of the medial wall and the lateral wall in patients with light proptosis (3–4 mm). In patients with intermediate proptosis (5-6mm), the anterior part of the medial wall and orbital fat (1-2ml) were resected additionally. In more severe cases, parts of the orbital floor and a maximum of orbital fat (2-3ml) were also removed to achieve a maximum effect. All orbital decompressions were performed by the same surgeon (S.M.).

### Computed tomography

**Fig 2:**
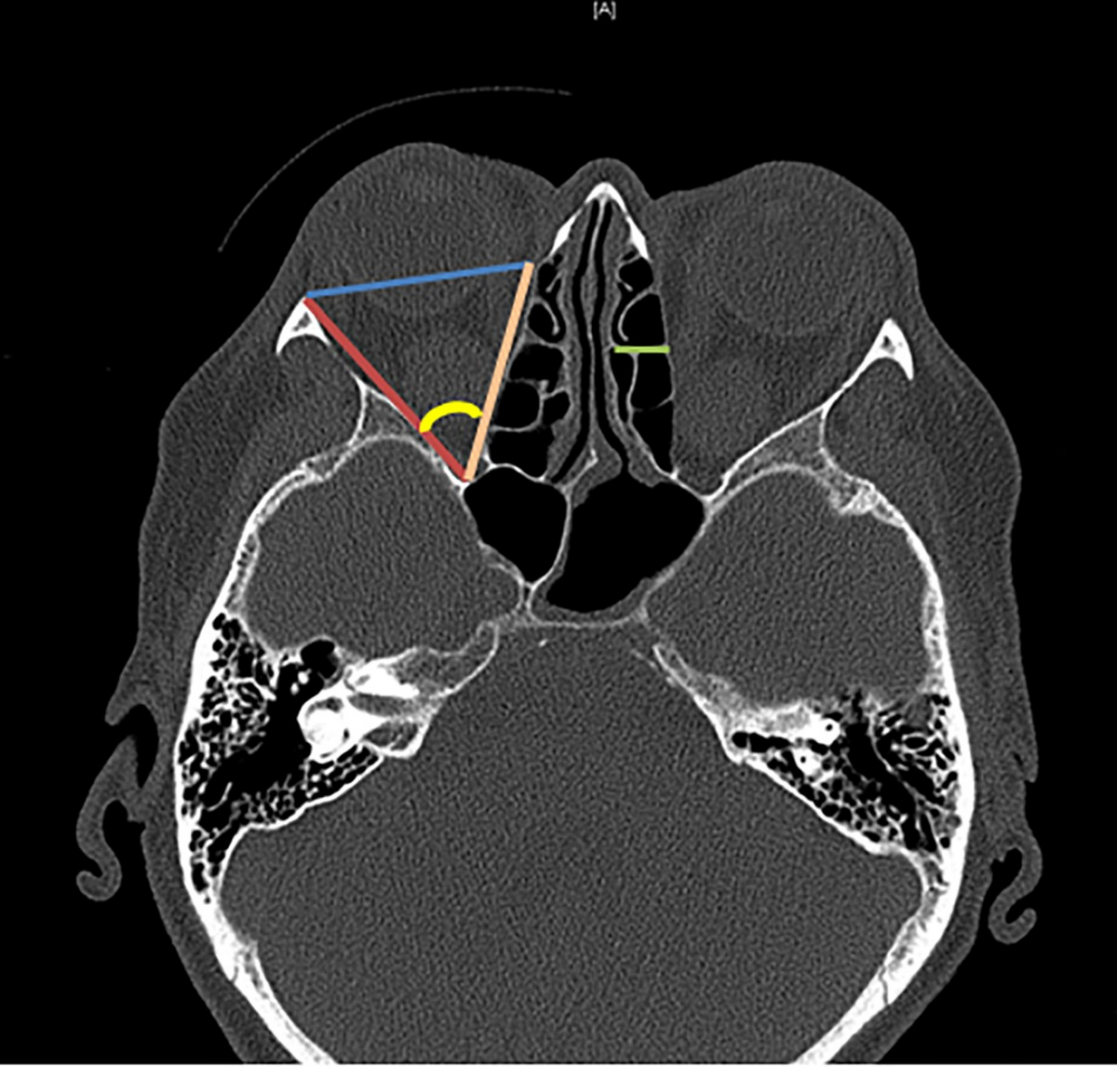
Pre- and postoperative computed tomography images were analyzed. The orbital area was measured as a triangle comprised of lateral (red) and medial orbital wall (orange), as well as orbital width (blue). Furthermore, the conus angle (yellow) and the depth of the ethmoid cavity were measured. Postoperative scans at the same level were used to measure length of medial and orbital defect and depth of medial tissue prolapse.

All patients underwent pre- and postoperative CT scans (1-3mm continuous slicing, bone windowing). Preoperative CT scans were performed for planning of surgery, whereas postoperative scans were performed to exclude bleedings and injuries of the skull base. The CT scans were used to analyze the anatomical features of the orbital morphology before and after surgery (see Fig 2). Axial scans at the level of the lens were used to determine the length of medial (orange line) and lateral (red line) walls, conus angle (yellow), depth of ethmoidal sinus (green line), orbital width (blue line) and the maximum horizontal muscle diameters of the superior muscle group (SMG, M. rectus superior and levator palpebrae superioris), the inferior rectus, medial rectus and lateral rectus in preoperative scans as described before [41, 42]. Postoperative scans at the same level were used to measure length of medial and orbital defect and depth of medial tissue prolapse. Toggling between pre- and postoperative CT scans allowed us to define resection borders as exactly as possible. All measurements were done by one investigator to secure homogeneity. Accuracy was checked by a second investigator who measured 10 CT scans, which were chosen randomly, separately. These measurements showed only minor differences (<0.3mm) and confirmed the reproducibility of the measurements. Orbital area was calculated as a triangle defined by medial and lateral wall and orbital width (comparable to the calculation of Campi et al. 2013) [43]. Furthermore, relation of the removed orbital wall to the wall length and the medial and lateral resected part of the wall length were computed.

### Statistical evaluations

For metric data, median values and range or the mean and standard deviation (SD±) were calculated and differences were evaluated with Student’s t-test (two-tailed) if D’Agostino-Pearson omnibus normality test showed normal distribution, if not, with Wilcoxon-Test. Fisher's exact test was used to evaluate group distributions of binary variables. Linear regressions for the uni- and bilateral group, as well as for all patients have been performed to test the association between morphological and outcome parameters. Multiple linear regression has been performed to assess further influential factors. Level of statistical significance was defined two-tailed as 2α<0.05. All calculations were performed with SPSS (IBM SPSS Statistics, Chicago, IL, USA, Version 22.0.0,) and Graph Pad Prism (Prism 6 for Windows, Software Inc., San Diego, CA, USA, Version 6.01). P-values are given descriptively without α-adjustment for multiple testing.

## Results

### Study population

Sixty-eight GO patients (mean age: 53.1 ±10.7 years) underwent balanced decompression surgery between 2011 and 2016 and fulfilled all inclusion criteria (see Table 1 for baseline characteristics). Of these, 16% (n = 11) underwent unilateral and 84% (n = 57) bilateral decompression, resulting in 125 operated orbits. The group consisted of 8 male and 60 female patients. DON was present in 12 patients (18%). 37 patients (54%) suffered preoperatively from diplopia of which 19 had diplopia in primary gaze.

**Table 1:** Baseline characteristics of study population.

	*n*	
Age (years)	68	53.1 ±10.7 [24,77]
Females	60	88.2%
Steroid therapy	59	86.8%
Irradiation	20	29.4%
Smoker	36	52.9%
Previous antithyroid treatments		
Radioiodine therapy	27	39.7%
Thyroidectomy	42	61.8%
Duration of thyroid disease (years)	68	2.8 [0.4, 40.0]
Duration of GO (years)	68	1.8 [0.2, 29.8]
DON	12	17.6%
Inactive GO	56	82.3%
Protrusion preoperatively (in mm)	125	24.5 ±2.8 [18, 31]
Horizontal deviation preoperatively (in PD)	68	0° [–40, 70]
Abduction preoperatively (in°)	125	40 [0–45]
Diplopia	37	54.4%
Diplopia in primary gaze	19	28%
BSV Score (0–100)	37	34.8 [0–90]

Unless otherwise stated data are means ±SD or proportions (%) or median [Range]; PD = prism diopters

BSV: Binocular single vision

### Surgical outcome

The surgical goal of reduction of proptosis could be achieved in most patients (mean reduction 5.3±2mm). While preoperatively proptosis was 25±3mm [range: 18–31], it was postoperatively significantly lower at 19±2mm [15–25] (see Table 2). Visual acuity was improved or stabilized in all patients suffering from DON. Patients with less than 5mm reduction had significantly less often new onset of diplopia compared to patients with ≥5mm reduction (13% vs. 56%, p = 0.02). Overall, patients with no preoperatively diplopia showed new onset of diplopia in primary gaze in 54.8% of the cases. The abduction and deviation worsened significantly (median: 5° and 10^Δ^; p<0.001) compared to baseline. Patients with previous orbital irradiation showed markedly less abduction deficit postoperatively compared to patients without prior irradiation without statistical significance (median 5° vs. 0°, p = 0.35).

**Table 2:** Postoperative patient characteristics.

	*n*		*P*
New onset of diplopia in primary gaze	17	54.8%	
Worsening of preexisting diplopia	18	48.6%	
Improvement of preexisting diplopia	5	13.5%	
BSV Score (0–100)	37	21.1 [0–100]	**<0.001**^**a**^
Protrusion (in mm)	125	19.2 ±2.3 [15–25]	**<0.001**^**a**^
Horizontal deviation (in PD)	68	10 [–20–50]	**<0.001**^**a**^
Abduction deficit (in°)	125	5 [–25–30]	**<0.001**^**a**^

Unless otherwise stated data are means ±SD or proportions (%) or median [Range]; a: Wilcoxon-Test

b: Fisher’s exact Test; BSV: Binocular single vision

### Orbital morphology

**Fig 3:**
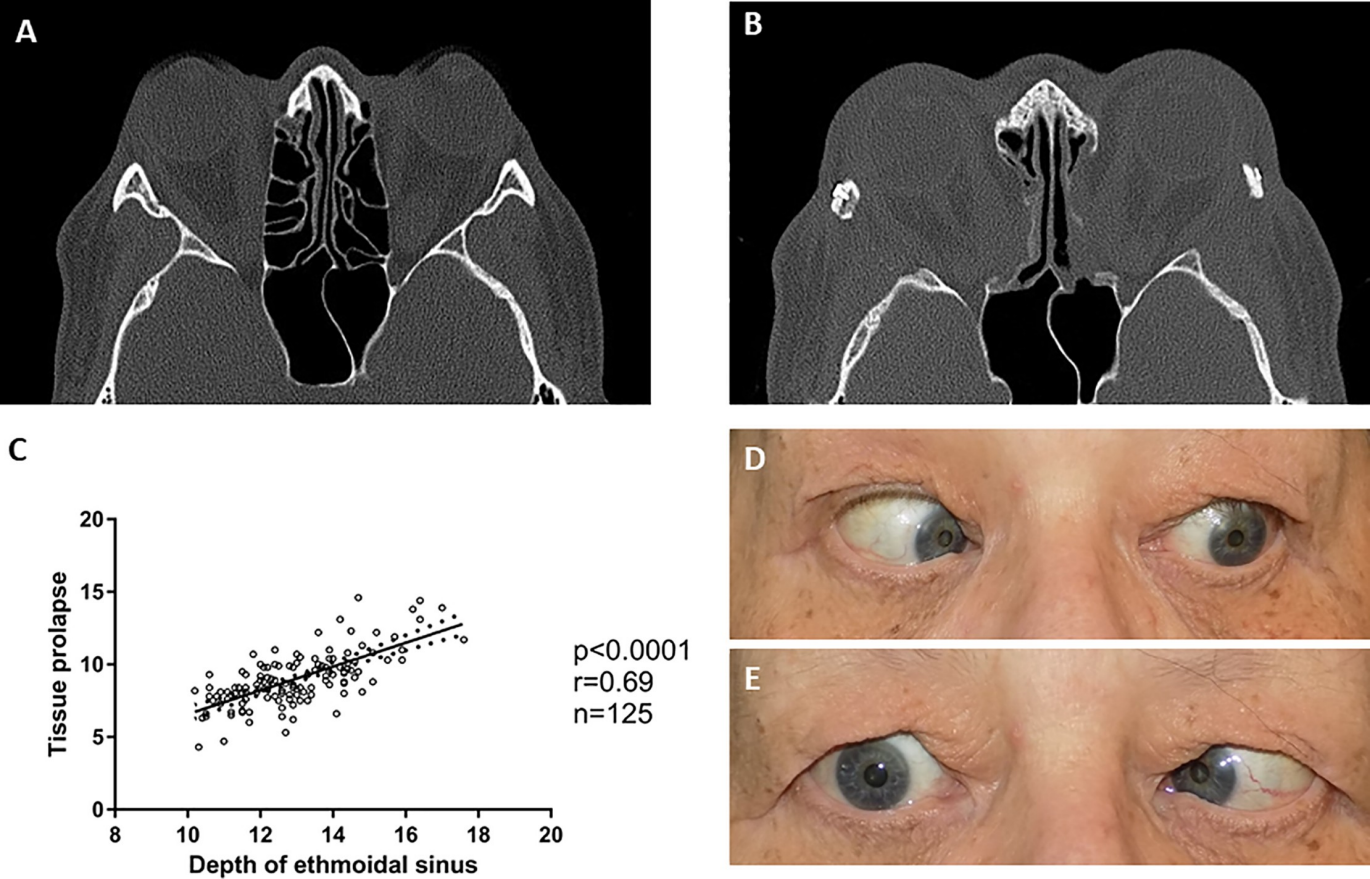
Preoperative CT scan of a patient with a deep ethmoidal cavity, with a high amount of tissue prolapse postoperatively **(A-B)** Linear regression showed a significant correlation between postoperative abduction deficit and tissue prolapse **(C)**, as exemplary shown in this postoperative images during left and right gaze **(D-E)**. Linear regressions were performed to predict proptosis reduction: The decrease of proptosis correlated significantly with conus angle (p = 0.03, r = 0.22), but not significantly with tissue prolapse, defect length, depth of ethmoidal sinus and the muscle diameter. Postoperative abduction deficit correlated significantly with the amount of tissue prolapse (p<0.01, r = 0.25) and orbital surface area (p = 0.01, r = 0.22). A multiple linear regression showed an even better correlation for both parameter combined (F(2,122) = 9,7; p<0.0001, r = 0.37). The maximum horizontal muscle diameter of SMG, IR and LR showed no significant correlation to the corresponding motility of the muscle. Adduction showed a significant correlation to the MR diameter (p<0.001, r = 0.10). Furthermore, a multiple regression was run to predict tissue prolapse from depth of the ethmoidal sinus and age (see Fig 3). These variables statistically significant predicted tissue prolapse, *F*(2,65) = 32.2, *p*<0.0001, r = 0.71. Durban-Watson statistic was used to check for autocorrelation, which was not the case. The duration of the disease showed no significant correlation with tissue prolapse and postoperative abduction deficit.

All CT images were of diagnostic image quality and analyzed for the orbital morphology (see Table 3). The measurements showed a wide variety, depending on the individual patients’ anatomy. Medial and lateral wall were 39±4 mm and 41±4 mm long. The ethmoidal sinus was 13±2 mm deep. Orbital surface area was 20±4 cm^2^ and the cone angle 50° (median). Postoperative measurements showed marked alterations: The lateral and medial walls were resected by 21mm [13–41] and 28mm [16–40] (median, range), respectively. The orbital tissue prolapsed into the ethmoidal sinus by 9mm [range: 4–15]. There was a significant correlation between tissue prolapse and depth of the ethmoidal sinus (see Fig 3).

**Table 3:** Orbital morphology.

	*n*	
Lateral orbital wall length (in mm)	125	40.5 ±3.8 [30, 50]
Medial orbital wall length (in mm)	125	38.5 ±4.3 [29, 50]
Depth of ethmoidal sinus (in mm)	125	13.0 ±1.6 [10, 18]
Orbital surface area (in cm^2^)	125	20.1 ±3.6 [11.4, 29.5]
Cone angle (in°)	125	50.2 [37, 71]
Horizontal muscle diameter		
SMG	125	6.8 ±2
IR	125	7.2±2
MR	125	7.0±2
LR	125	4.8±1
Total	125	25.9±5
Postoperative measurements		
Lateral defect length (in mm)	125	21.4 [13–41]
Medial defect length (in mm)	125	28.4 [16–40]
Soft tissue prolapse (in mm)	125	8.8 [4–15]

Unless otherwise stated data are means ±SD or median [Range]

SMG: Superior muscle group (M. rectus superior and levator palpebrae superioris)

IR: M: inferior rectus; MR: M. rectus medialis; LR: M. rectus lateralis

## Discussion

In this retrospective single-center study, a detailed analysis of the orbital morphology before and after bony decompression surgery in patients with Graves’ orbitopathy was successfully accomplished. Our results show that CT morphology should be carefully evaluated to estimate the risk of postoperative abduction deficit. However, two-dimensional CT measurements could not safely predict the proptosis reduction.

### Surgical outcome

A satisfying amount of proptosis reduction could be achieved in most patients. The amount of proptosis reduction is higher than in comparable studies using similar techniques [25, 33, 44–46]. However, protrusion of orbital soft tissues into the ethmoidal sinus led often to impaired motility, misalignment and consequently to diplopia as described by others before [18, 20, 34]. This might be due to the more severely afflicted patient cohort in out trial with higher initial proptosis. Therefore, the surgeon aims to achieve the highest amount of decompression by maximal bony resection. Furthermore, we observed in patients with higher proptosis often a larger prolapse probably due to the higher intraorbital pressure. The amount of increase in deviation (10^Δ^ vs. 9.6^Δ^) and decrease in motility (both 5°) in our cohort is in concordance with other trials [47]. The existing practice to wait with extraocular muscle and eyelid surgery until after decompression is thus reassured. A previous trial could show that orbital irradiation before orbital decompression shows a protective effect for ocular motility deterioration [48]. The same tendency was present in our cohort. Furthermore, we could show that postoperative abduction deficit can be predicted by the amount of tissue prolapse and orbital surface area with multiple linear regression. The tissue prolapse in turn can be predicted by the depth of the ethmoidal sinus. Therefore, patients with deep ethmoidal sinus (≥14.9mm) and large orbits should be informed that they are at a greater risk for postoperative motility disorders, especially, if they are of geriatric age. In these patients, the amount of bony resection should be contemplated especially carefully. In contrary, for patients with small ethmoidal sinus (≤10.9mm) it might be an option to consider an applicable extent of orbital fat resection to assure a proper amount of proptosis reduction, since orbital tissue cannot expand as much into the ethmoidal sinus in these patients.

In general, preoperative patient information should include that a higher proptosis reduction (≥5mm) leads to a higher risk of postoperative diplopia. If possible, the intended proptosis reduction can be chosen accordingly to minimize the occurrence of diplopia, as diplopia can decrease the quality of life drastically [7]. If higher proptosis reduction is necessary, misalignment can be corrected with extraocular muscle surgery later, even in patients with large esotropia [22, 49]. Therefore, fear for diplopia should not limit the orbital decompression, especially in patients with DON. Still, sometimes more than one strabismus surgery is needed to accomplish fusion in primary gaze and patients should be informed about the long duration of the rehabilitation process to prevent false presumptions [18, 22].

### Orbital morphology

The detailed morphological analysis of the orbit before and after bony decompression surgery was successfully accomplished. Our results are comparable to previously published CT measurements of the orbit. Cone angle was here from 37° up to 70°, which is comparable to measurements from Kamer et al. (2010), who found 39–66° in 140 orbits with 3D measurements [35]. Horizontal muscle diameters were slightly higher compared to Dagi et al. (2011), which is explainable by the more severely afflicted patients in our cohort. Overall, our measurements in routine CT scans could not sufficiently predict the amount of proptosis reduction. One reason is probably the limitations of two-dimensional measurements: Curved orbital walls are measured with straight measurements [50]. Newer, more complex techniques in three-dimensional scans are able to map the real orbit and not only an approximation [28, 36, 37, 51]. However, even they might not be sufficient, since the prolapse of orbital tissue during decompression surgery shows great individual variety. Derived from this and the low coefficients of correlation in terms of proptosis reduction, we hypothesize, that besides the osseous structures of the orbit, the properties of the orbital tissue are of major importance for the surgical outcome. Especially, the degree of fibrosis and the muscle volume might enable prediction of proptosis reduction. Future studies should consider these parameters to enable virtual surgery planning. In this retrospective analysis, the muscle volume could unfortunately not be evaluated reasonably. Still, we analyzed the muscle diameter of the 4 muscle groups, which showed no significant correlation with proptosis reduction. To summarize, an individualized treatment plan for decompression surgery will require, besides exact measurement of the bony orbit, measurements of the orbital tissue, especially of the muscles. However, even muscles volumes might not be sufficient to predict the prolapse since the grade of fibrosis is hard to analyze preoperatively. Therefore, it seems rather unlikely that we will be able to predict the proptosis reduction and postoperative motility disorders precisely. A detailed planned extent of orbital wall resection will only be helpful if surgery is intraoperatively controlled. For this task intraoperative navigation seems appropriate [52, 53].

### Conclusion

Patients with higher proptosis reduction suffer more often from postoperative diplopia. Routine CT measurements of the orbit can support the surgeon in planning of the operative procedure. Deep ethmoidal sinus and larger orbital surface areas seem to be a risk factor for postoperative abduction deficit. Patients with these characteristics should be operated with special care. However, proptosis reduction cannot be predicted with routine CT scans. Advancements in three-dimensional imaging and virtual planning of orbital decompression surgery might lead to a safer and more efficient surgery in the future.
