# Correction: Influence of orbital morphology on proptosis reduction and ocular motility after decompression surgery in patients with Graves' orbitopathy

**DOI:** 10.1371/journal.pone.0220821

**Published:** 2019-07-31

**Authors:** The PLOS ONE Staff

The affiliation for the eighth author is incorrect. Kerstin Stähr is not affiliated with #2 but with #3: Department of Oto-Rhino-Laryngology, Head and Neck Surgery, University Hospital Essen, Essen, Germany. The publisher apologizes for the error.
